# Circumferential and radial myocardial strain in cardiomyopathy patients with and without left bundle branch block

**DOI:** 10.1186/1532-429X-11-S1-P14

**Published:** 2009-01-28

**Authors:** Yuchi Han, Jonathan Chan, Idith Haber, Dana C Peters, Peter J Zimetbaum, Warren J Manning, Susan B Yeon

**Affiliations:** 1 BIDMC, Boston, MA USA; 2 University of Chicago, Chicago, IL USA

**Keywords:** Cardiac Resynchronization Therapy, Left Bundle Branch Block, Mechanical Dyssynchrony, Right Bundle Branch Block, Depressed Left Ventricular Function

## Introduction

Electrical dyssynchrony associated with prolonged QRS duration is a commonly used criterion to select symptomatic heart failure patients for cardiac resynchronization therapy (CRT). However, there is concern that electrical dyssynchrony criterion is inadequate as 30–40% of patients do not respond to CRT [1]. Studies have shown that assessment of mechanical dyssynchrony may be a better predictor of response [2]. Most of the studies have assessed mechanical dyssynchrony in the longitudinal axis of myocardial motion [3]. There is limited data on the assessment of short axis mechanical dyssynchrony in humans. We sought to examine the relationship between electrical and mechanical dyssynchrony in mid-ventricular short axis using CMR tagging in patients with depressed left ventricular function.

## Methods

22 patients with NYHA class II to III heart failure were studied, including 12 patients with dilated cardiomyopathy (DCM) (age 60 ± 9 years, 83% male, ejection fraction (EF) 28 ± 9%) and 10 patients with ischemic cardiomyopathy (ICM) (age 63 ± 8 years, 80% male, EF 30 ± 5%). Ten healthy adult subjects (age 37 ± 12 years, 50% male, EF 60 ± 4%) served as controls. CMR studies were performed on a 1.5 T Philips Achieva MR scanner (Philips HealthCare, Best, NL), equipped with a 5-element cardiac coil. Breath-hold ECG-gated tagged CSPAMM cine images at the mid-papillary muscle level were obtained. Scan parameters include spiral readout with 8 interleaves, 9 ms acquisition window, TR/TE/flip angle = 25 ms/3.6 ms/25°, FOV = 320 mm, 10 mm slice thickness with 5 mm tag spacing, temporal resolution 25–35 ms, spatial resolution 2.5 × 2.5 × 10 mm. A customized software program (Cardiotool), written in MATLAB (MathWorks, Natick, MA), was used for semi-automated analysis of peak circumferential (εc) and peak radial strain (εr) [4].

## Results

**Figure 1:**
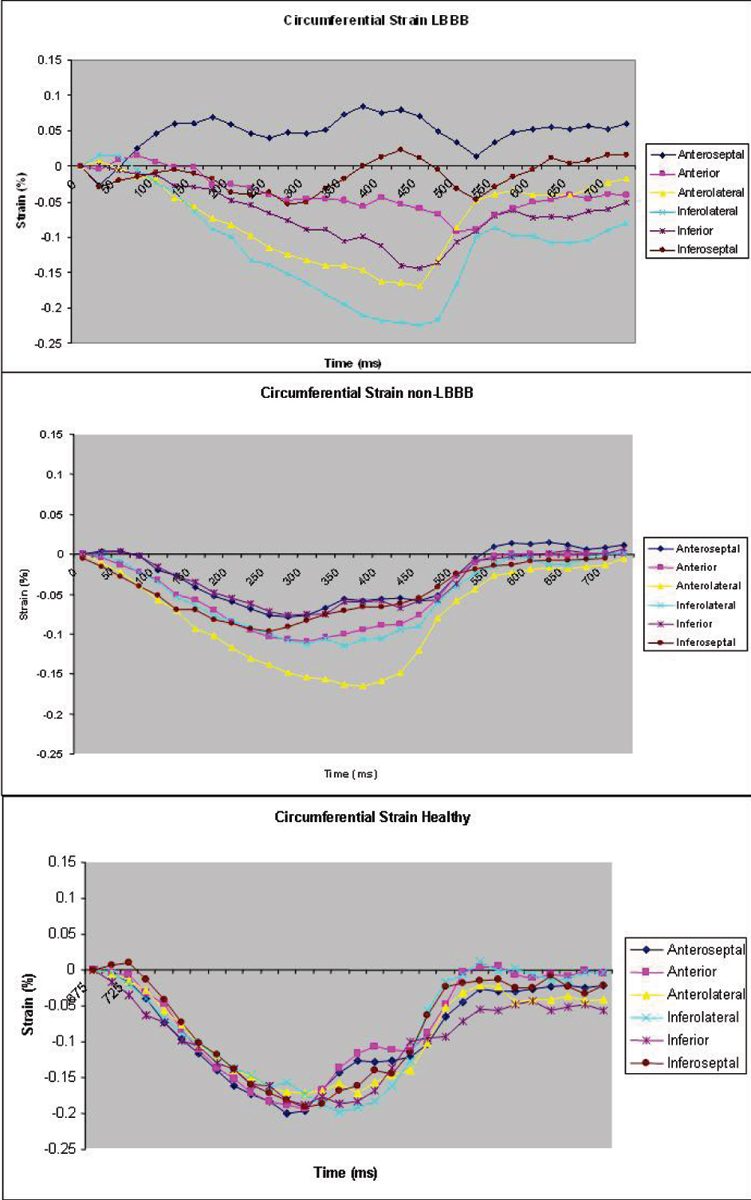
**Representative circumferential myocardial strain in cardiomyopathy patients with LBBB, non-LBBB, and healthy controls**.

**Figure 2:**
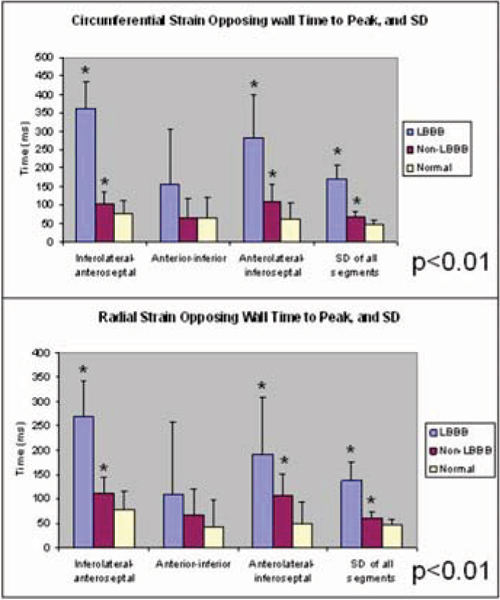
**LBBB patients have more mechanical dyssynchrony than non-LBBB patients**.

Thirteen patients with DCM (n = 8) and ICM (n = 5) had a left bundle branch block (LBBB) with a mean QRS duration of 161 ± 15 ms. The remaining nine patients had either normal QRS duration, non-specific interventricular conduction delay, or right bundle branch block with a mean QRS duration of 108 ± 18 ms. The control subjects had a mean QRS duration of 94 ± 11 ms. All patients with LBBB showed an initial negative εc in the anteroseptal segment, quickly followed by positive εc reflecting dyskinesis of the septum (as shown in Figure 1). In patients with LBBB, there was significant dysynchrony of contraction as indicated by greater opposing wall (inferolateral wall to anteroseptum) delays and standard deviations (SD) in time to peak εc and εr for all six wall segments (p < 0.001) (Figure 2). All patients without LBBB and all normal subjects showed negative εc in all segments throughout systolic contraction (Figure 1). Patients with cardiomyopathy showed reduced magnitude of εc and εr (p < 0.002).

## Conclusion

In patients with heart failure, a LBBB pattern with marked increase in QRS duration manifests as a specific contractile pattern with marked dyskinesis of the interventricular septum. Mechanical dyssynchrony and reduction in myocardial circumferential and radial strain are identified and quantified using a semi-automated method. This study identifies the relationship between electrical and mechanical circumferential and radial dyssynchrony in patients with dilated as well as ischemic cardiomyopathy.
